# Unexpected Air Following Ankle Injury

**DOI:** 10.1016/j.acepjo.2025.100092

**Published:** 2025-03-13

**Authors:** Lyra B. Olson, Jakara Morgan, Malcolm R. DeBaun, Timothy Scott Peterson

**Affiliations:** 1 Duke University Medical Scientist Training Program, Duke University School of Medicine, Durham, North Carolina, USA; 2 School of Medicine, University of South Carolina Greenville, Greenville, South Carolina, USA; 3 Department of Orthopedic Surgery, Duke University School of Medicine, Durham, North Carolina, USA; 4 Department of Emergency Medicine, Duke University School of Medicine, Durham, North Carolina, USA

**Keywords:** ankle dislocation, subcutaneous gas

## 1 Case Presentation

**Figure 1:**
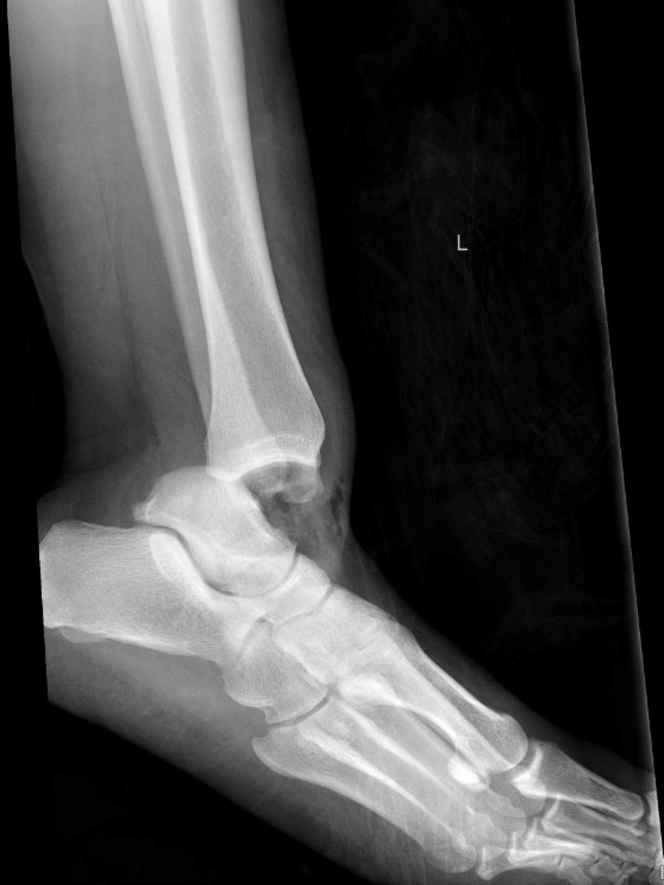
Initial ankle radiograph demonstrating locules of gas in soft tissue.

**Figure 2:**
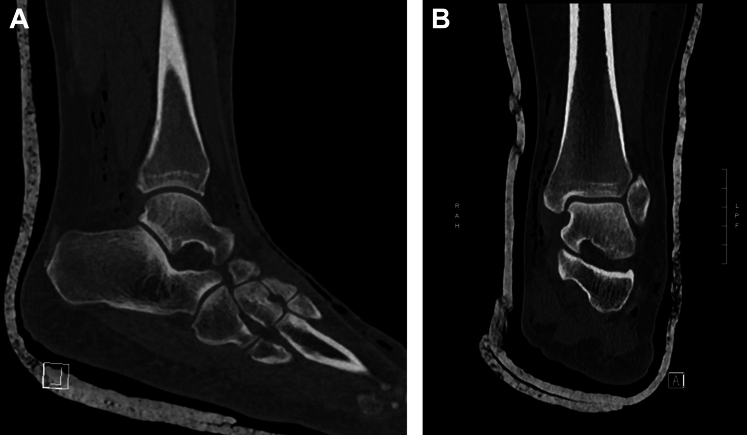
(A and B) Postreduction computed tomography with gas tracking in subcutaneous space.

**Figure 3:**
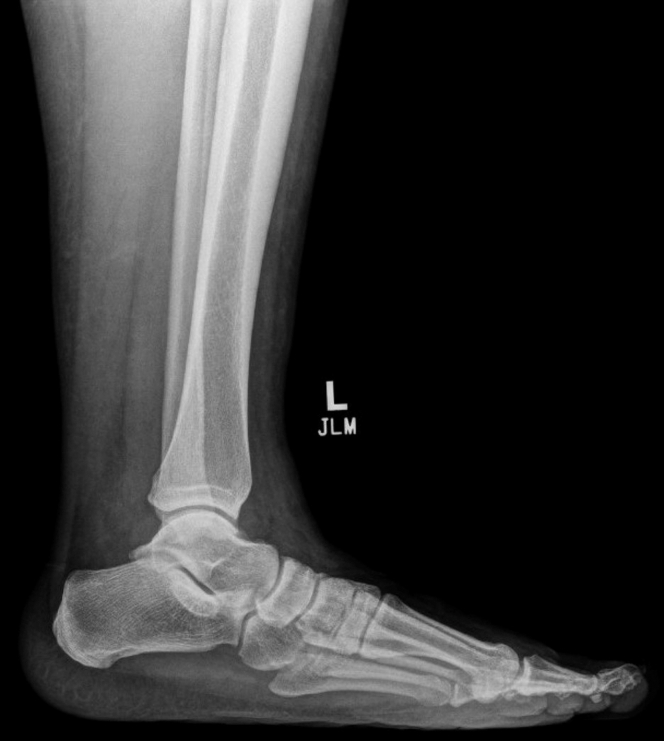
Lateral radiograph at 10-day follow-up.

A 41-year-old woman presented to the emergency department with ankle pain after a fall down 3 steps. Examination revealed a closed, grossly deformed, neurovascularly intact ankle with a shallow 0.5 cm laceration over the lateral proximal foot. Radiographs showed medial posterior tibiotalar dislocation with locules of gas tracking within the soft tissue of the lateral ankle (Fig 1). Repeat imaging after hematoma block, relocation, and splinting demonstrated extensive subcutaneous emphysema of the ankle and foot along with intraarticular gas (Video 1 and Fig 2). Radiology expressed concern for traumatic arthrotomy, and a single dose of cefazolin was provided, but further doses were deferred in the absence of clinical concern for open fracture. X-ray at follow-up in the clinic 10 days later demonstrates a well-reduced, congruent mortise with no notable subcutaneous air (Fig 3).

**Video 1:**
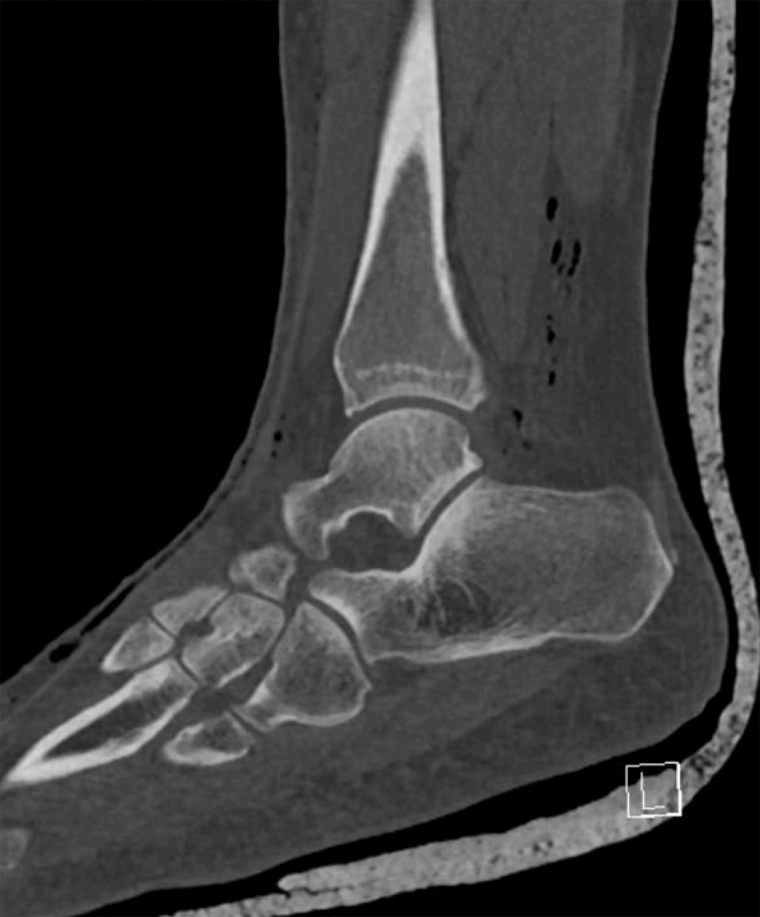
Postreduction computed tomography.

## 2 Diagnosis: Benign Subcutaneous Emphysema

Subcutaneous emphysema in an extremity generally raises concern for open fracture or a gas-producing infectious process. This rare case demonstrates incidental subcutaneous air, initially localized near the joint and later dispersed throughout subcutaneous space following manual manipulation for relocation and splinting. Two mechanisms for emphysema formation likely contributed: from outside the body via a 1-way valve of air entry though disrupted skin^1^ or from gas released from body fluids in the sudden pressure drop created by the vacuum of tissue disruption at the moment of injury.^2^ This condition is usually self-limited and managed conservatively. This case emphasizes the utility of physical examinations in guiding treatment courses to avoid unnecessary antibiotic exposure.

## Funding and Support

By *JACEP Open* policy, all authors are required to disclose any and all commercial, financial, and other relationships in any way related to the subject of this article as per ICMJE conflict of interest guidelines (see www.icmje.org). The authors have stated that no such relationships exist.

## Conflict of Interest

All authors have affirmed they have no conflicts of interest to declare.
